# The communication between patient relatives and physicians in intensive care units

**DOI:** 10.1186/s12871-017-0388-1

**Published:** 2017-07-17

**Authors:** Faruk Cicekci, Numan Duran, Bunyamin Ayhan, Sule Arican, Omur Ilban, Iskender Kara, Melda Turkoglu, Fatma Yildirim, Ismail Hasirci, Adnan Karaibrahimoglu, Inci Kara

**Affiliations:** 1 Department of Anesthesiology, Selcuk University, Medical Faculty, Alaadin Keykubat Yerleskesi, Konya, Turkey; 2 Scool of Foreing Languages, Selcuk University, Konya, Turkey; 3 Department of Journalism, Selcuk University, Communication Faculty, Konya, Turkey; 4 Department of Anesthesiology, Konya Numune State Hospital, Konya, Turkey; 5 Department of Internal Medicine, Gazi University, Medical Faculty, Ankara, Turkey; 6 Department of General Surgery, Konya Training and Education Hospital, Konya, Turkey; 7 Department of Biostatistics, Suleyman Demirel University, Faculty of Medicine, Konya, Turkey

**Keywords:** Communication, Intensive care unit, Professional-family relations, Relatives, Surveys and questionnaire

## Abstract

**Background:**

Patients in intensive care units (ICUs) are often physically unable to communicate with their physicians. Thus, the sharing of information about the on-going treatment of the patients in ICUs is directly related to the communication attitudes governing a patient’s relatives and the physician.

This study aims to analyze the attitudes displayed by the relatives of patients and the physician with the purpose of determining the communication between the two parties.

**Methods:**

For data collection, two similar survey forms were created in context of the study; one for the relatives of the patients and one for the ICU physicians. The questionnaire included three sub-dimensions: informing, empathy and trust. The study included 181 patient relatives and 103 ICU physicians from three different cities and six hospitals.

**Results:**

Based on the results of the questionnaire, identification of the mutual expectations and substance of the messages involved in the communication process between the ICU patients’ relatives and physicians was made. The gender and various disciplines of the physicians and the time of the conversation with the patients’ relatives were found to affect the communication attitude towards the patient. Moreover, the age of the patient’s relatives, the level of education, the physician’s perception, and the contact frequency with the patient when he/she was healthy were also proven to have an impact on the communication attitude of the physician.

**Conclusion:**

This study demonstrates the mutual expectations and substance of messages in the informing, empathy and trust sub-dimensions of the communication process between patient relatives and physicians in the ICU. The communication between patient relatives and physicians can be strengthened through a variety of training programs to improve communication skills.

## Background

Patients in intensive care units are physically unable to give information about their health history. In this situation getting the anamnesis of a patient in treatment is directly related to the communication attitudes governing the interaction between the patient relatives and the physician. Communication is based on source, message and receiver [1]. The communication source is usually the physician in the health units such as intensive care units. The conversation between the patient relatives and the physician is the message, and the patients relatives is the recipient. In order for the communication process to function properly, the physician and the patients’ relative must attribute the same meaning to the message. It is known that attitudes are the driving forces behind behavior, and also attitudes can be defined as the likely behavior that an individual is expected to display in a given situation, event or phenomenon [2]. Nevertheless, attitudes can be learned and managed our actions [3]. In particular, one of the vital criteria of similarity in developing common attitudes and orienting behavior in specific areas, such as health, is that communication has an effect on communication towards engagement and attitude [1, 2]. However, current studies indicate that the quality of communication between the relative and the physician is often poor [4, 5]. Furthermore, most physicians are not even aware of this shortcoming [6]. The studies concerning the relatives were mainly about the end-of-life family conference [7–9]. Moreover, there were limited scale for communication between the patient relatives and the physician [10, 11]. This study was intended to analyze the attitudes governing the interaction between the patient relatives and the physician using the two-part questionnaire form that inquire the communication skills of patient relatives and the physician.

## Methods

This study was conducted in three cities in Turkey (Konya, Ankara and Bursa) between March 1 and September 1, 2015 in the ICUs of six hospitals (state, university and private hospitals). The researcher obtained the approval of the Medical Ethics Committee of Selcuk University, Faculty of Medicine (Ethics No: 2015/98).

The researcher identified the number of patients that stayed in the intensive care unit (ICU) for 3 days or longer in the 6-month period when the study was conducted in order to determine the number of attitude questionnaires needed for the study. It was found that there were 710 patients who stayed in the ICU for 3 days or longer. The necessary approval and informed consent forms were obtained from the relatives of the 181 patients. The total number of intensive care beds in the participating hospitals was 87. Regarding the 181 patient relatives who were included in the study, the distribution according to the hospital was determined on the basis of the ratio of the number of intensive care beds in that hospital to the total intensive care beds of all the hospitals involved in the study (Table 1). These relatives volunteered to participate in the study, spoke Turkish, were literate and had a conversation with the ICU physician at least three times. Also, 103 physicians who worked on the ICUs agreed to participate in the study.

**Table 1:** The patients’ relatives and physician numbers to be taken to the pilot study according to intensive care beds numbers of hospitals

Hospitals of Study-City	İntensive care unit bads	The patients’ relatives included in the study	The physician included in the study
Konya Numune State hospital-Konya	30	62 (34.5%)	31 (30.0%)
Selcuk University, Medical Faculty Hospital-Konya	8	17 (9.2%)	19 (18.4%)
Private Medicana Hospital-Konya	15	31 (17.2%)	8 (7.8%)
Konya Training and Education Hospital-Konya	12	25 (13.8%)	17 (16.5%)
Gazi University, Medical Faculty Hospital-Ankara	14	29 (16.1%)	16 (15.5%)
Bursa Training and Education Hospital-Bursa	8	17 (9.2%)	12 (11.7%)
Over all	87	181 (100%)	103 (100%)

Excluded from the study sample were: the patients’ relatives that were younger than 18 years; relatives who accompanied patients that stayed less than 3 days in the ICU, relatives who spoke to the physician less than 3 times, and those who did not want to participate in the study. The ICU physicians that had spoken to the patients’ relatives less than 3 times and did not wish to participate in the study were also excluded from the study.

For this study, we prepared two similar survey forms for the patients’ relatives and the physicians. These forms included questions on the socio-demographical features of the patients’ relatives and the physicians, and questions to determine the effectiveness of the communication between the patients’ relatives and the physicians in the ICU. For the latter, 3 sub-dimensions were developed: informing, empathy and trust. Individual questionnaires were developed for the physicians and the relatives of the patients. The researcher created the questionnaire based on the patient-physician communication questionnaire that was developed by Curtis et al. (2004) for chronic obstructive pulmonary disease patients in serious condition [12]. But the questionnaires created are specific to this research. The sample size was calculated based on a total of 710 cases in 6 months using simple random sampling. To initiate the content validity process, the survey forms were distributed to five experts. After evaluating the results from the experts’ assessments, a Content Validity Index was developed. Next, a pilot study was conducted to secure the validity and reliability of the surveys. The pilot study composed of smaller groups determined using simple random sampling (Table 1).

The questions were reviewed and the questionnaire was finalized after making observations in the ICUs. In the process of developing the questionnaire, a Communications professor was on hand daily to hear the complaints of the patients, following the approval of the patient; the total observation time was about 30 h.

All statistical analyses were performed using the Statistical Package for Social Science (SPSS, 20.0 SPSS FW, SPSS Inc., Chicago, IL., USA). Descriptive statistics were applied to analyze the responses to the socio-demographic items. Categorical variables are presented as frequencies and percentages; numerical variables are shown as median (first and third quartiles) in the tables since the Kolmogorov-Smirnov test revealed an anomaly in the distribution of the numerical variables. Because of the lack of normal distribution, non-parametric tests were used in comparison analyses. The second part of the survey, which serves to measure the attitudes of the patient relatives and the physicians, was developed as a 5-point Likert scale with responses ranging from 1 (Never) to 5 (Always). Total item scores were calculated by adding the points given for all of the items. However, the scores of questions 3, 4 and 13 in the physician’s language and communication sub-dimension were inverted (inverted Likert scale), since the statements in these questions were structured negatively in contrast to the statements in the other questions. The Mann-Whitney U test was applied for comparing two independent groups, while the Kruskal-Wallis test was applied for multiple independent groups, using the pairwise comparison technique in cases of significant differences between groups. In the pilot study, the Cronbach’s Alpha value for reliability was calculated and the test-retest method was applied to reinforce the reliability, accompanied by performance of the Wilcoxon Signed Rank test for repeated measures. A Principal Component Analysis with Varimax rotation was performed to obtain the factors with percentage of cumulative loading squares for validity. The models were regressed by automatic linear modelling with forward selection to control for confounding factors over informing, empathy and trust dependent variables. In all analyses, a *p* < 0.05 value was considered to be a statistically significant result, and 5% was accepted as type-I error.

A total of 183 patients’ relatives were found to be sufficient when type-I error was 5%, the power was 80%, the general population N was 710, the satisfaction rate 80%, and the effect size (d) 0.05.

## Result

### The patients’ relatives

Table 2 presents the results of the 5-Point Likert type communication attitude questionnaire that was administered to a total of 181 patient relatives.

**Table 2:** The scoring percentages of responses by the patients’ relatives to questions about informing, empathy and trust sub-dimensions on a 5-Point Likert type communicative attitude scale

QUESTIONNAIRE ITEMS FOR THE PATIENTS’ RELATIVES					
	Always %	Very Often %	Some times %	Rarely %	Never %
SUBDIMENSION OF INFORMING					
1- I believe that the frequency of being informed about my patient is sufficient.	59.1	16.0	14.4	9.4	1.1
2- After speaking to the physician, I still feel that I am informed insufficiently.	29.8	9.9	22.7	17.1	20.4
3- I believe I am learning about the medical situations regarding my family member in the most comprehensive way.	62.4	23.2	7.7	4.4	2.2
4- I receive all possible information about my family member whenever I speak to the physician.	71.3	20.4	3.9	3.3	1.1
5- The physician uses language that I can understand.	69.1	17.1	8.3	4.4	1.1
6- I would like to receive the medical information about my family member while I am next to the patient himself/herself.	38.7	19.3	7.7	16.0	18.2
7- Physicians respond to all my questions.	64.6	19.9	8.8	4.4	2.2
8- Physicians have difficulty giving bad news.	38.1	31.5	12.2	8.3	9.9
SUBDIMENSION OF EMPATHY					
9- I believe the physician cares about my family member.	72.9	15.5	0.0	1.1	10.5
10- I believe that the physician cares about me as the patient relative.	71.8	14.9	2.2	1.1	9.9
11- When I have a problem with the physician, I make an effort to think about it calmly.	40.9	33.1	12.7	9.9	3.3
12- It makes it easier for me to communicate when the physician approaches me in a friendly manner.	69.1	15.5	7.7	2.2	5.5
16- When the physician tells me what to do about my family member, this makes things easier for me.	75.7	18.2	1.7	4.4	0.0
17- I believe that my physician treats everyone equally.	73.5	9.9	9.9	3.3	0.0
18- ICU physicians are friendly and smiling.	56.9	24.9	11.6	6.6	0.0
19- ICU physicians have an understanding attitude.	66.3	18.8	9.4	6.1	2.2
20- I believe that I receive the necessary support from the physicians.	61.9	20.4	9.4	6.1	2.2
SUBDIMENSION OF TRUST					
21- I feel peaceful after speaking to the physician.	63.9	21.1	9.9	5.0	0.0
22- I feel nervous while speaking to the physician.	18.2	19.9	16.0	21.5	24.3
23- While speaking to the physician, I trust in what he/she says.	78.5	15.5	2.2	3.9	0.0
24- I can access my family member’s physician whenever I need to.	51.9	16.6	16.6	6.6	8.3
25- If a problem occurs regarding my family member, the physician is responsible for solving it.	30.4	26.5	8.8	8.8	25.4
26- ICU physicians are very reassuring.	64.6	25.4	6.6	3.3	0.0

The questionaire assesses the communicative skills of patients’ relatives and the physicians

The comparison of socio-demographic data by informing, empathy and trust on the attitudes towards communication questionnaire of the patients’ relatives is shown in Table 3.

**Table 3:** The compare of socio-demographic data on the communication towards attitude questionaire of the patients’ relatives according to the informing, empathy and trust sub-dimensions

	*N*	INFORMING		EMPATY		TRUST	
		Median (25th-75th Percentile)	*p*	Median (25th–75th Percentile)	*p*	Median (25th–75th Percentile)	*p*
Gender							
Male	116	37 (21–44)	0.378	41 (18–44)	0.087	25 (16–30)	*0.011*
Female	65	36 (20–45)		40 (30–45)		24 (12–28)	
Education							
No education	8	41 (34–41)^a^	*0.006*	40.5 (40–45)	*0.015*	26 (26–26)	*0.003*
Primary school	56	37 (26–44)^b^		41 (32–45)^a^		24 (17–29)	
Middle school	25	36 (27–41)^a,b^		41 (18–44)		22 (16–28)	
High school	58	37 (21–45)		41 (31–45)		26 (19–30)^a^	
University	34	37 (20–42)		38 (24–45)^a^		22 (11–29)^a^	
Descriptions of physicians							
Legal-technical consultant	74	37 (21–43)^a^	*0.036*	40 (24–45)^a^	*0.041*	25.5 (16–30)	0.181
Advisor	50	37 (20–41)		41 (18–45)^b^		22 (11–29)	
Friend	12	38.5 (31–42)		42 (34–45)		26 (22–29)	
Protector	32	41 (29–45)^a^		45 (24–45)^a,b^		24 (16–29)	
Others	13	34 (32–41)		40 (34–45)		25,823–28)	
Age groups							
< 35	54	33 (20–45)^a,b^	*0.001*	37 (18–45)^a,b^	*0.001*	22 (11–30)	0.131
35–50	65	37 (27–43)^a^		42 (31–45)^a^		25 (17–29)	
> 50	62	39 (26–44)^b^		42 (32–45)^b^		26 (16–29)	
How close the patients’ relatives were to the patient							
Spouses	31	40 (31–42)		41,824–45)		26 (19–27)^a^	
Children	104	37 (22–44)		41 (18–44)		24 (11–29)^b^	
Sister/Brother	17	36 (29–41)	0.082	42 (37–45)	0.332	25 (16–30)	*0.043*
Grandson	14	41 (28–45)		37 (24–44)		22 (22–30)	
Parents	5	40 (26–43)		40 (31–45)		23 (19–26)	
Cousin/distant relative	10	37.5 (34–41)		40.5 (36–45)		20.5 (19–22)^a,b^	
The frequency of patient relatives’ seeing the patients before they were taken to the ICU							
More than once a day	41	37 (20–43)^a^		41 (30–44)^a^		25 (11–29)^a^	
Once a day	102	38 (28–44)		41 (18–44)^b^		25 (16–30)^b^	
Once in every 2 or 3 days	32	35.5 (21–45)^b^	*0.015*	40 (31–44)^c^	*0.002*	22.5 (16–28)	*0.009*
Once in a week or less	6	45 (31–45)^a,b^		37 (24–37)^a,b,c^		28 (26–28)^a,b^	
The frequency of visits to the patients in the ICU by patient relatives							
Everyday	86	37.5 (20–44)	0.159	41 (18–45)^a^	*<0.001*	26 (11–29)^a,b^	*0.001*
Once in every 2 or 3 days	60	36 (21–43)		40 (24–45)^b^		22 (16–30)^a,c^	
Once a week	12	38.5 (29–45)		37 (32–43)^c^		21 (19–28)^d^	
Less than once a week	23	37 (31–41)		26 (24–45)^abc^		26 (14–29)^bcd^	
The duration of how long the patient relatives spoke to the physicians							
1–2 min	43	37 (20–459	0.289	38 (24–45)^a^	*0.001*	24 (11–28)	0.079
5 min	93	37 (26–44)		40 (18–45)^b,c^		25 (17–29)	
10 min	44	35.5 (27–43)		42 (32–45)^a,b^		23 (16–29)	
> 10 min	15	37 (35–41)		45 (41–45)^c^		22 (21–30)	
The situations that relieved the stress of patient relatives*							
Speaking with the doctor							
Yes	121	37 (20–43)	0.487	41 (24–45)	*0.002*	26 (11–30)	*0.001*
No	60	36 (21–44)		38 (18–44)		22 (16–29)	
Being with the patient							
Yes	93	37 (21–45)	*0.001*	40 (18–45)	*0.001*	24 (11–29)	0.537
No	88	37.5 (26–45)		42 (32–459		24.5 (17–30)	
Praying							
Yes	70	36.5 (20–43)	*0.004*	40 (18–45)	0.245	24 (11–30)	0.855
No	111	38 (21–45)		41 (24–45)		25 (16–29)	
Getting good news							
Yes	104	37 (22–45)	0.508	41 (18–45)	0.742	24 (11–29)	0.594
No	77	37 (21–43)		41 (24–45)		25 (16–30)	
The characteristics of ICU physicians that were important to patient relatives*							
Getting good news							
Yes	83	37 (29–44)	*0.001*	42 (24–45)	*0.001*	24 (16–29)	0.308
No	98	36 (20–45)		40 (18–45)		24 (11–30)	
Giving accurate information							
Yes	128	39 (28–44)	*0.042*	43 (18–45)	*0.002*	25 (11–30)	*0.009*
No	53	37 (22–45)		40 (24–45)		22 (16–29)	
Having a sympathetic attitude							
Yes	37	37 (20–42)	*0.016*	40 (24–45)	0.324	26 (11–29)	*0.003*
No	144	37 (21–43)		41 (18–43)		24 (16–31)	
Detailed medical explanation							
Yes	54	37 (18–42)	0.569	41 (18–45)	0.760	25 (19–29)	*0.008*
No	127	37 (20–45)		40 (24–45)		24 (11–30)	
Interest and relevance							
Yes	73	37 (20–43)	0.522	41 (24–45)	0.871	24 (11–30)	0.676
No	108	37 (21–45)		41 (18–45)		24.5 (16–29)	

*Multiple answers were given

^a,b,c,d^The results found statistical difference in group was shown in the same letter

The results found statistical difference in group was shown as italicize data

The regretion analysis of socio-demographic data on the communication towards attitude questionaire of the patients’ relatives according to the informing, empathy and trust sub-dimensions is shown in Table 4.

**Table 4:** The scoring percentages of responses by physicians to questions about informing, empathy and trust subdimensions on a 5-Point Likert type communicative attitude scale

QUESTIONNAIRE ITEMS FOR THE PHYSICIANS					
	Always %	Very Often %	Some times %	Rarely %	Never %
SUBDIMENSION OF INFORMING					
1- I believe that the frequency of informing the patient relative about my patient is sufficient.	35.0	47.6	15.5	1.9	0.0
2- After speaking to the patient relative, I still feel like I have provided insufficient information.	14.6	52.4	26.2	2.9	3.9
3- I believe I describe the medical condition of my patient in the most comprehensive way.	26.2	54.4	14.6	4.9	0.0
4- When I speak to a patient relative, I give all the information about the patient.	25.2	58.3	12.6	3.9	0.0
5- I use language that patient relatives can understand when I am telling them about the medical situations related to their patients.	44.7	50.5	0.0	4.9	0.0
6- I would like to give the medical information about my patient next to the patient himself/herself.	8.7	22.3	19.4	29.1	20.4
7- I would like to respond to all the questions that patient relatives ask.	29.1	42.7	23.3	3.9	1.0
8- Physicians have difficulty giving bad news.	39.8	37.9	16.5	5.8	0.0
SUBDIMENSION OF EMPATHY					
9- I believe that I care about my patient.	59.2	32.0	4.9	1.9	0.0
10- I believe that I care about the patient relatives in addition to the patients.	40.8	52.4	4.9	1.9	0.0
11- When I have a problem with the patient relative, I try to think in a calm manner.	23.3	47.6	13.6	11.7	3.9
12- When patient relatives have a friendly approach, this makes it easier for me to build a close relationship.	35.9	45.6	17.5	1.0	0.0
13- When my directions about my patient are followed, this makes things easier for me.	58.3	39.8	0.0	1.9	0.0
14- I believe that, as a physician, I treat everyone equally.	46.6	48.5	4.9	0.0	0.0
16- ICU physicians are friendly and smiling.	6.8	41.7	40.8	10.7	0.0
17- ICU physicians have an understanding attitude.	19.4	59.2	20.4	1.0	0.0
18- I believe that, as a physician, I give the required support.	27.2	62.1	10.7	0.0	0.0
SUBDIMENSION OF TRUST					
19- I feel peaceful after speaking to the patient relative.	15.5	47.6	29.1	6.8	1.0
20- I feel nervous while speaking to the patient relative.	6.8	17.5	48.5	21.4	5.8
21- I trust the patient relative while speaking to him/her.	5.8	35.0	28.2	24.3	6.8
22- The patient relative can access me whenever he/she needs to see me about the patient.	22.3	55.3	13.6	6.8	1.9
23- If a problem occurs about my patient, I am responsible for it.	6.8	8.7	23.3	34.0	27.2
24- I would like to foster confidence as an ICU physician.	67.0	33.0	0.0	0.0	0.0

The questionaire assesses the communicative skills of the patients’ relatives and the physicians

There was a difference in the trust sub-dimension between the genders of patients’ relatives. There were differences in the informing, empathy and trust sub-dimensions among the education levels of the patients’ relatives (*p* = 0.006, *p* = 0.015 and *p* = 0.003, respectively). There were also differences in the informing and empathy sub-dimensions according to descriptions of physicians by patient relatives (*p* = 0.036 and *p* = 0.041; respectively) as well as the informing and empathy sub-dimensions among the age groups of patients’ relatives (*p* < 0.001). There was a difference in the trust sub-dimension by the closeness of the relatives to the patient (*p* = 0.043). Also, there were differences within the informing, empathy and trust sub-dimensions by the frequency of patients’ relatives seeing the patients before they were taken to the ICU (*p* = 0.010, *p* = 0.007 and *p* = 0.012; respectively), and in the empathy and trust sub-dimensions by the frequency of visits to the patients in the ICU by patient relatives (*p* < 0.001).

There was a difference in the empathy sub-dimension by the duration patient relatives’ conversations with the physicians (*p* < 0.001). Regarding situations that relieved the stress of patient relatives, “speaking with the doctor” was different in the empathy and trust sub-dimensions (*p* = 0.002 and *p* < 0.001); “being with the patient” was different in the informing and empathy sub-dimensions (*p* = 0.001 and *p* < 0.001), and “praying” was different in the informing sub-dimension (*p* = 0.004).

Regarding the characteristics of ICU physicians that were important to the patients’ relatives, the “giving good news” group was different in the informing and empathy sub-dimensions (*p* = 0.001 and *p* < 0.001), the “giving accurate information” group was different in the informing, empathy and trust sub-dimensions (*p* = 0.042, *p* = 0.002 and *p* = 0.009, respectively), and the “having a sympathetic attitude” group was different in the informing and trust sub-dimensions (*p* = 0.016 and *p* = 0.003).

### The physician

Table 5 presents the results of the 5-Point Likert type questionnaire that was administered to 103 ICU physicians.

**Table 5:** The compare of socio-demographic data on the communication towards attitude questionaire of the physicians according to the informing, empathy and trust sub-dimensions

	*n*	*INFORMING*		*EMPATHY*		*TRUST*	
		*Median (25th–75th Percentile)*	*p*	*Median (25th–75th Percentile)*	*p*	*Median (25th–75th Percentile)*	*p*
Gender							
Male	56	34 (27–42)	0.192	37 (29–42)	*0.007*	20 (16–25)	0.085
Female	47	36 (26–44)		39 (28–45)		22 (14–28)	
Age groups							
< 35	74	35 (26–42)	0.715	37 (26–44)	0.777	20 (12–28)	0.073
35–50	24	33 (26–42)		38 (29–44)		22 (17–27)	
> 50	5	35 (31–37)		38 (35–40)		19.5 (17–22)	
Descriptions of physicians							
Legal-technical							
Consultant	56	37 (34–41)	0.227	39.5 (37–41)	0.183	23 (22–24)	0.153
Advisor	19	36 (27–42)		38 (32–45)		22 (17–28)	
Friend	10	36 (26–42)		39 (30–45)		20 (16–27)	
Protector	11	35 (28–41)		38 (29–40)		20 (16–27)	
Others	7	36 (27–41)		37 (26–40)		19 (12–23)	
The specialties of the ICU physicians							
Anesthesiology	57	34 (26–42)	0.051	36 (29–37)^a^	*0.009*	21 (16–28)^a^	*<0.001*
Pulmonologist	14	35 (28–42)		38 (29–45)		18 (12–22)^ab^	
Cardiovascular surgery	9	36 (29–37)		37 (32–39)^b^		20 (16–22)^c^	
Internal medicine	8	37 (36–42)		40 (40–44)^ab^		23 (22–24)^bc^	
Surgery	3	38 (33–38)		37 (37–38)		22 (22–22)	
Emergency medicine	12	35 (28–37)		39 (29–45)		20 (17–23)	
How close of the relative being informed by the physician was to the patient							
Spouses	38	34 (28–42)	0.348	38 (29–45)	0.342	20 (16–25)	*0.035*
Children	55	35 (26–42)		37 (26–44)		22 (12–27)^a^	
Parents	5	37 (28–41)		37 (29–45)		17 (16–28)	
Cousin and other distant relatives	5	35 (32–40)		38 (35–40)		19.5 (17–22)^a^	
The frequency of patient relatives’ speaking the physician							
More than once a day	22	36 (28–41)	0.287	39 (34–45)	0.272	21.5 (16–28)	0.528
Once a day	66	35 (26–42)		37 (28–45)		20 (12–28)	
Once in every 2 or 3 days	11	34 (27–38)		38 (31–42)		22 (20–23)	
Once in a week or less	4	33 (30–34)		35 (35–35)		19 (17–19)	
The duration of how long the ICU physicians spent speaking to patient relatives							
1–2 min	13	32 (27–37)	0.129	33 (26–37)^ab^	*0.001*	19 (12–23)	0.141
5 min	60	35 (28–42)		38 (29–45)^a^		21 (16–28)	
10 min	25	35 (26–41)		40 (29–45)^b^		22 (20–22)	
> 10 min	5	41 (31–41)		39 (34–39)		20 (20–21)	
The ICU physician characteristics that were important to patient relatives*							
Accurate information							
Yes	56	35 (27–42)	0.224	38.5 (26–45)	0.205	20 (12–18)	0.081
No	47	35 (26–42)		37 (29–45)		22 (16–27)	
Sympathetic attitude							
Yes	28	36 (26–41)	0.350	38.5 (29–45)	0.637	22.5 (17–28)	*<0.001*
No	75	35 (27–42)		37 (26–45)		22 (16–27)	
Provision of medical support							
Yes	11	36 (32–40)	*0.032*	39 (33–44)	0.487	22 (20–28)	*0.001*
No	92	35 (27–42)		37 (26–44)		20 (12–27)	
Interest and relevance							
Yes	47	36 (27–41)	0.117	38 (29–45)	0.241	20 (16–28)	0.448
No	56	34 (26–42)		37 (26–44)		20.5 (12–27)	
Confidence							
Yes	76	35 (26–42)	0.368	38 (26–45)	0.079	20 (12–28)	0.617
No	27	35 (27–41)		36 (29–41)		21 (16–23)	

*Multiple answers were given

^a,b,c^The results found statistical difference in group was shown in the same letter

The results found statistical difference in group was shown as italicize data

Table 6 presents the comparison of socio-demographic data regarding the sub-dimensions of informing, empathy and trust on the attitude toward communication questionnaire for physicians.

**Table 6:** The regretion analysis of socio–demographic data on the communication towards attitude questionaire of the patients’ relatives according to the informing, empathy and trust sub–dimensions

	*INFORMING*		*EMPATHY*		*TRUST*	
	β	*p*	β	*p*	β	*p*
Gender						
Female					−1.233	*0.009*
Education						
Middle school	−1.792	*0.010*	3.566	*<0.001*	−1.759	*0.001*
University						
Descriptions of physicians						
Advisor					1.347	*0.004*
Friend					1.366	*0.033*
Protector					1.366	*0.033*
Age groups						
< 35	−3.861	*<0.001*	−4.134	*<0.001*	−1.872	*<0.001*
How close the patient relatives were to the patient						
Spouses						
Grandson						
Cousin/distant relative	1.828	*0.013*				
The frequency of patient relatives’ seeing the patients before they were taken to the ICU						
Once in a week or less	7.170	*<0.001*			1.052	*0.014*
Once a day					8.996	*<0.001*
Once a week					8.996	*<0.001*
The frequency of visits to the patients in the ICU by patient relatives						
Once a week						
Everyday	1.363	*0.038*				
Once in every 2 or 3 days					3.201	*<0.001*
Once a week						
The duration of how long the patient relatives spoke to the physicians						
1–2 min						
5–10 min					−1.974	*<0.001*
5–10 min			4.046	*0.001*		
> 10 mins						
The situations that relieved the stress of patient relatives*						
Being with the patient						
No	2.440	*0.003*	−2.654	*<0.001*		
Praying						
Yes	8.917	*0.004*				
No	8.962	*0.004*	3.369	*<0.001*		
The characteristics of ICU physicians that were important to patient relatives						
Getting good news						
No	−3.765	*<0.001*	−2.165	*0.001*		
Giving accurate information						
No	−1.952	*0.019*				
Having a sympathetic attitude						
No						
Detailed medical explanation	2.779	*0.007*				
No					−1.579	*0.001*

* Multiple answers were given

For Informing AIC (akaike information criterion) =512.89, Accuracy 45.2%; Empathy AIC = 516.78, Accuracy 50%; Trust AIC = 373.72, Accuracy 47.2%

The results found statistical difference in group was shown as italicize data

The regretion analysis of socio-demographic data on the communication towards attitude questionaire of the patients’ relatives according to the informing, empathy and trust sub-dimensions is shown in Table 7.

**Table 7:** The regretion analysis of socio-demographic data on the communication towards attitude questionaire of the patients’ relatives according to the informing, empathy and trust sub-dimensions

	*INFORMING*		*EMPATHY*		*TRUST*	
	**β**	*p*	β	*p*	β	*p*
Descriptions of physicians						
Advisor	−2.572	*0.041*				
Friend			2.078	*0.016*		
Legal-technical	−1.825	*0.016*				
Consultant	−2.504	*0.002*				
Advisor						
The specialties of the ICU physicians						
Pulmonologist						
Cardiovascular surgery					−2.864	*<0.001*
Internal medicine	4.971	*<0.001*	−3.247	*<0.001*		
How close of the relative being informed by the physician was to the patient						
Children	3.157	*0.010*			1.498	*0.002*
The duration of how long the ICU physicians spent speaking to patient relatives						
1–2 min	−2.204	*0.037*	−3.878	*<0.001*	−2.260	
5–10 min						*0.002*
The ICU physician characteristics that were important to patient relatives						
Provision of medical support						
No	−2.493	*0.036*				
Confidence						
No						
Provision of medical support						
No			−1.841	*0.012*	−2.418	*0.003*

For Informing AIC (akaike information criterion) =260.08, Accuracy 30%; Empathy AIC = 243.32, Accuracy 37.2%; Trust AIC = 170.80, Accuracy 45.7%

The results found statistical difference in group was shown as italicize data

There was a difference in the empathy sub-dimension for ICU physicians by gender. There were also differences in the empathy and trust sub-dimensions by their specialties (*p* = 0.009 and *p* < 0.001). There was a difference found in the trust sub-dimension by the closeness of the relative that was informed by the physician to the patient (*p* = 0.035). Regarding the ICU physician characteristics that were important to patient relatives, “sympathetic attitude” was different in the trust sub-dimension (*p* < 0.001), and “provision of medical support” was different in the informing and trust sub-dimensions (*p* = 0.032 and *p* = 0.001).

## Discussion

Through the analysis of responses on the three sub-dimensions of the attitudes towards communication questionnaire, this study has demonstrated the mutual expectations and the substance of the messages in the communication process between the relatives of the patients in the ICU and the attending physicians. As part of the study, suggestions have been presented on how to improve management of the sub-dimensions mentioned and on meeting expectations.

Communicative skill is one of the most important factors within the relationship between patients’ relatives and physicians. The communication between physicians and patient relatives is not just about exchanging information about epicrisis. It is also about a relationship between two persons, especially concerning how well they communicate. The fundamental elements of this communication are credibility, context, content, clarity, continuity and consistency, channels, and capability of audience [13]. This study found that male patient relatives have more trust in physicians. This result is not surprising considering that female patient relatives can be more emotional.

In the present day, it is easier to access information through the internet and other means. This can lead to an increase in the number of university graduate patients and the patient relatives that read about and thoroughly understand diseases and treatments. These patients and relatives may make demands, express dislike of the staff or physician, and criticize the treatment method [14]. This study determined that the higher the education level of patient relatives was, the less they thought that information from the physicians was sufficient. Similarly, the levels of empathy with the physicians, and the level of trust in the physicians were reduced as education levels of the patient relatives increased.

In the relations where patients are passive and physicians are assertive, physicians are seen as a “father figure” who always considers the patients’ best interests. However, the changes in the concepts of disease and health in the twentieth century, the differences in the identities of physicians (because of specialties and sub-specialties), and increased technology in medicine with the emergence of the “right to health” concept, have led to conflicts between the values of patients and physicians. These conflicts are also the result of the autonomy of patients, and their desire to have a role in medical decisions [15]. Yet, patient relatives continue to see physicians as their “protectors”. This study also found that the patient relatives who regarded physicians as their protectors received more information from physicians, and had a deeper empathy for physicians.

The relevant literature mainly focuses on the communication between young patients and physicians [16, 17].

This study found that young patients’ relatives (35 years and younger) are less informed by physicians, and they empathized less with them.

There were no studies in the literature about how frequently relatives visited the patient, and what effect this had on their communication with the physicians. In this study, the relatives that saw their family members more frequently before hospitalization thought they were informed insufficiently, and had a lack of trust in the physicians. Yet they empathized with the physicians more.

The time patient relatives spend with physicians is very short, but it can be the most important time of the day. Most patient relatives stated that, during this time, physicians usually did not supply sufficient information, their conversation was interrupted continuously, and they were not able to ask important questions [9, 18, 19]. This study showed that when the daily communication lasted for 10 min or longer, patient relatives and physicians found it easier to empathize with each other.

The effectiveness of the communication between physicians and patient relatives in the health care system is determined by socio-economic conditions, education level, religion, attitudes regarding ethics, ethnic and cultural background, previous experiences, perception of physicians and expectations [20]. Hunsucker et al. [21] found that trust and being well-informed were the most important needs for families. These needs were followed by being close to the patients, and receiving comfort and support. In this study, the patient relatives who were relieved after speaking to the physicians empathized more with them and trusted them more. Moreover, the patient relatives who wanted to receive good news from the physicians thought that they were better informed, and empathized more strongly with the physicians. Yet the patient relatives that were relieved when they were with their family members thought that they were not informed sufficiently, and had a weaker empathy with the physicians. The patient relatives that were relieved by praying did not value the information they received from the physicians. It is estimated that the limited period of visits to ICUs prevented most patient relatives from getting answers to all of their questions.

Most patients in ICUs are unable to cooperate with their physicians. For this reason, the families of the patients in ICUs experience a high level of emotional stress [22]. Other studies determined that patient relatives emphasized the importance of communication, stating that information about patients was more than just emotional support [23, 24]. In this study, the patient relatives that cared about being given accurate information stated that they were informed better, and had greater empathy and trust in the physicians. The patient relatives that cared about the friendliness of physicians trusted their physicians more.

Relevant studies have shown that varied factors including the length of daily working hours, workload, and lack of professional experience increase burnout levels. This caused physicians to have less spare time for themselves and for social activities. This can decrease the quality of life [25]. These negatives may eventually reflect on their relationship with their patients. It is commonly agreed in the relevant literature that female physicians inform patients and patient relatives better than male physicians, empathize more, and engage in casual conversation more with patients [10, 26, 27]. This study also found that female physicians empathized more with patient relatives. This is probably due to the fact that male physicians generally use their left-brain functions (e.g. problem solving) while female doctors mainly use their right brain functions including those used in inter-personal relationships [28].

The relationship between physicians and patients is between two persons who are not equal. The physician knows much more about diagnosis and treatment. Therefore, trust is very important in these relationships [29]. While some patients desire to use their autonomy and have full control over medical decisions, others prefer that their physicians make all the decisions. However, patients benefit from treatment only if they have a trusting relationship with their physicians [30]. This study found that specialists in internal medicine empathize better with their patients and build a more trusting relationship than cardiovascular surgeons and anesthesiologists do. This probably results from the patient-focused approach used by primary care or internal medicine physicians as well as a more frequent use of communicative skills. These skills and approaches are not commonly used by the physicians that are specialized in anesthesia or radiology. They might be somewhat distant from patients and patient relatives.

Patient relatives might expect physicians to be friendly and be informed about everything. These behaviors and attitudes may foster trust in the patient relative-physician relationship. These feelings may also be easily damaged in a negative situation. When there are further developments in the diagnosis and treatment process, patient relatives may feel desperation, hopelessness and pessimism, in addition to feelings distress and anxiety. This may lead to excessive sadness and depression. This situation may develop into what is called a post intensive care syndrome-family. A variety of studies have shown a high prevalence of anxiety and depression in patient relatives [31, 32]. Major anxiety and depression probably affect understanding, comprehension and the ability to communicate. In this study, the patient relatives were spouses or parents of the patient, which enabled building stronger trusting relationships with physicians.

In varied publications, it is noted that physicians can contribute as much as 60 to 70% to the communication between physicians and patient relatives [33]. A noteworthy feature regarding physician and patient relative conversations is that relatives mostly perceive these conversations to be short. Varied studies have demonstrated that a sufficient length for the patient relative-physician conversation is at least 10 minutes [34, 35]. This study found that conversations with patient relatives lasting at least 10 minutes create a stronger empathy.

Gaining the trust of patient relatives in the first conversation is very important in terms of communication. The most important factors regarding first impressions are what physicians do and do not say, and how they say it [27, 31]. Past studies that were conducted with families from different cultures have found that the primary needs of family members are trust and being informed [21, 26, 36–38]. Molter and Leske stated that the most important needs of patient relatives were feeling that there was hope for the patients, being informed sufficiently and honestly, and believing that the hospital staff was providing good care [27, 38]. This study found that the physicians who were able to display a sympathetic attitude (Sympathy is the ability to compassionately identify with a person’s emotional state) were able to build stronger, trusting relationships with patient relatives. The physicians who believed that good medical care was important in their relationships with patient relatives provided better information, and built a stronger, trusting relationship.

This study has some limitations. Firstly, the researchers did not have an available questionnaire that could assess the communication between patients’ relatives and physicians. This made it obligatory to create a brand-new attitude questionnaire. The creation of the attitude questionnaire was a very challenging process since the content of the conversation gets more diverse as more people are included, and communication is a quite expansive field of study. However, the researchers used a variety of resources to create the questionnaire, and consulted with physicians and communication researchers. Secondly, communication with health professionals is mainly limited to the communication between patients and physicians, and there are few published articles about the communication between patient relatives and physicians.

## Conclusion

This study made an attempt to reveal the mutual expectations and the substance of the messages by analyzing the informing, empathy and trust sub-dimensions of the communication process between the relatives of the patients in the intensive care unit and physicians.

After all, the communication between patient relatives and physicians is the communication between two parties, and it requires an exchange of information, mutual support, respect and trust. The physicians are professionals who need to communicate with patient relatives, and solve the communication problems. The communication between patient relatives and physicians can be improved through a variety of training programs to improve communication skills since attitudes can be learned and managed our actions.

## Abbreviations

ICU: Intensive Care Unit
SPSS: Statistical Package for Social Science
